# Molecular Pathogenesis of B-Cell Posttransplant Lymphoproliferative Disorder: What Do We Know So Far?

**DOI:** 10.1155/2013/150835

**Published:** 2013-04-14

**Authors:** J. Morscio, D. Dierickx, T. Tousseyn

**Affiliations:** ^1^KU Leuven, Translational Cell and Tissue Research, Leuven, Belgium; ^2^UZ Leuven, Department of Hematology, University Hospitals KU Leuven, Leuven, Belgium; ^3^UZ Leuven, Department of Pathology, University Hospitals KU Leuven, Leuven, Belgium

## Abstract

Posttransplant lymphoproliferative disorder (PTLD) is a potentially fatal disease that arises in 2%–10% of solid organ and hematopoietic stem cell transplants and is most frequently of B-cell origin. This very heterogeneous disorder ranges from benign lymphoproliferations to malignant lymphomas, and despite the clear association with Epstein-Barr Virus (EBV) infection, its etiology is still obscure. Although a number of risk factors have been identified (EBV serostatus, graft type, and immunosuppressive regimen), it is currently not possible to predict which transplant patient will eventually develop PTLD. Genetic studies have linked translocations (involving *C-MYC, IGH, BCL-2*), various copy number variations, DNA mutations (*PIM1, PAX5, C-MYC, RhoH/TTF*), and polymorphisms in both the host (*IFN-gamma, IL-10, TGF-beta, HLA*) and the EBV genome to B-cell PTLD development. Furthermore, the tumor microenvironment seems to play an important role in the course of disease representing a local niche that can allow antitumor immune responses even in an immunocompromised host. Taken together, B-cell PTLD pathogenesis is very complex due to the interplay of many different (patient-dependent) factors and requires thorough molecular analysis for the development of novel tailored therapies. This review aims at giving a global overview of the currently known parameters that contribute to the development of B-cell PTLD.

## 1. Introduction

Posttransplant lymphoproliferative disorder (PTLD) is the most severe complication of solid organ and hematopoietic stem cell transplantation and occurs in 2%–10% of posttransplant patients. The presentation of PTLD is highly variable and ranges from benign lymphoproliferations to overt lymphoma. In the majority of cases, PTLD is of B-cell origin [1] rather than T-cell origin [2] and presents most commonly as extranodal non-Hodgkin lymphoma (NHL) of which diffuse large B-cell lymphoma (DLBCL) is most frequent. Currently, the etiology of PTLD is not clear although 60%–80% of the cases have been associated with Epstein-Barr Virus (EBV) infection, which has been put forward as one of the main factors contributing to PTLD development. It has been speculated that the remaining EBV-negative PTLD cases are related to other viral infections (e.g., Human Herpes virus 8, Cytomegalovirus [3, 4]), are caused by hit-and-run infection [5] or chronic antigen stimulation by the graft or are coincidentally occurring lymphoproliferations similar to lymphomas in immunocompetent hosts [6]. The risk of lymphoma development posttransplantation is 20%–120% higher compared to the normal population, but currently it is not possible to predict which transplant recipients will ultimately develop PTLD [7]. In case of malignant B-cell PTLD, different types of lymphoma can arise. This variability can be partly explained by the complex development of B-cells: aberrations in each step of the developmental process can eventually contribute to lymphomagenesis. Analogously to NHL arising in an immunocompetent population, B-cell PTLD can be subdivided according to the cell of origin into germinal center B-cell-derived and activated B-cell-derived subtypes [8, 9].

This review aims at giving a general overview of the pathogenetic mechanisms that underlie B-cell PTLD. Because of the rarity of PTLD, insight into disease development and progression is currently limited. Most probably, EBV predisposes infected B-cells to uncontrolled proliferation which may result in the accumulation of (epi)genetic aberrations. Furthermore, the microenvironment of the transformed lymphocyte together with the genetic background of the individual provide a particular environment that could further promote lymphomagenesis.

## 2. PTLD Is a Multifactorial Disease

Despite its obscure pathogenesis, a number of commonly accepted risk factors for PTLD development have been identified. EBV-naive individuals lacking cellular immunity to EBV are susceptible to graft-mediated EBV infection resulting in early-onset PTLD (within 12 months following transplantation). This population mainly comprises children [10]. Analogously, patients undergoing myeloablative hematopoietic stem cell transplantation (HSCT) or individuals with a chronically high EBV viral load following transplantation are prone to early-onset PTLD [11], although the latter is still debated [12]. Regarding HSCT, PTLD development also greatly varies according to the procedure and ranges from 0.5% (for *HLA*-matched noncomplicated transplants) to 25% (for T-cell-depleted highly immunosuppressed transplants that lack T-cell-mediated anti-EBV immune responses) [13]. Furthermore, the risk of developing PTLD has been associated with the type of solid organ graft: grafts containing a substantial amount of lymphoid tissue (e.g., small intestine) and/or requiring an intensified immunosuppressive regimen (e.g., heart) are important predisposing factors for PTLD [14, 15]. Following transplantation, patients usually receive a combination of induction and long-term maintenance immunosuppressive therapy. Studies have associated several immunosuppressive drugs with increased (cyclosporin, tacrolimus, antithymocyte globulin, etc.) or decreased risk (antimetabolites azathioprine and mycophenolate mofetil, anti-CD52 antibody alemtuzumab) for PTLD development. However, apart from the type of drug, the combination schemes, cumulative intensity, and duration of administration also influence the risk [16]. Overall, PTLD development following solid organ transplantation (SOT) is estimated at 1%–5% (our group reported an average incidence of 2.12% in a mixed adult and pediatric population [1]), with a highest incidence for intestinal and multivisceral transplants (5%–20%) followed by lung and heart transplants (2%–10%) and lowest for renal and liver transplantation (1%–5%) [16, 17]. The occurrence of PTLD is highest within the year following transplantation and is strikingly higher for HSCT (210 cases/10.000/year) than SOT (22/10.000/year), falling dramatically to 5/10.000/year for both HSCT and SOT after the first year [18, 19]. Lucas et al. suggested that low levels of anti-EBV cytotoxic T-lymphocyte precursors following HSCT are associated with the higher risk of early-onset PTLD. By 6 months following transplantation, the level of cytotoxic T-lymphocytes is restored in most patients, and after 12 months, T-cell function is normalized [20]. 

90% of PTLD arising following SOT are derived from the postgerminal center host lymphocytes suggesting a role for chronic B-cell stimulation by the graft and endogenous EBV reactivation [21]. In case of PTLD post-HSCT, lymphoma arises most commonly from the donor lymphocytes and may result from graft-versus-host disease. It is not yet clear what the implications of both are in terms of survival, but a difference in onset and EBV status has been detected [22]. Solid organ transplants with host-derived PTLD tend to be at higher risk for persistent and recurrent disease [23], whereas donor-derived PTLD is characterized by onset already in the first months following transplantation [20, 22]. It is not known what causes PTLD to develop early after transplantation in some patients and late in others. One possible explanation is that in the latter, early-onset PTLD lesions remain subclinical for a long time. Overall, early-onset PTLD is characterized by involvement of lymph nodes or the allograft whereas late-onset PTLD presents more commonly extranodally [24].

Reported mortality rates of PTLD range from 25% to 60% [25] and overall survival is highly variable [26–29] due to the heterogeneity of the disease and the high rate of non-PTLD-related deaths (because of infection and other malignancies) [26]. 

## 3. PTLD Presentation Ranges from Benign to Malignant Lymphoproliferations

The World Health Organisation (WHO) discriminates three types of morphological lesions assumed to represent different stages in the pathogenesis of PTLD: benign polyclonal early lesions, polymorphic PTLD, and malignant monoclonal monomorphic PTLD [30]. It is assumed that monomorphic lymphoma gradually arises from early and polymorphic lesions but this process is not well understood. Early lesions (E-PTLD) are characterized by reactive proliferations and cannot be discriminated from an inflammatory response in an immunocompetent setting. Mass lesions lacking tumor cells arise most frequently around 3 months following transplantation in tonsils or lymph nodes where the normal architecture is maintained. Three types of early lesions are recognized: plasmacytic hyperplasia-like PTLD, infectious mononucleosis-like PTLD, and florid follicular hyperplasia (FFH). So far, it is not clear what implications these histological subtypes have in terms of prognosis, but they all consist of benign polyclonal lymphoproliferations that mostly regress when the immunosuppressive regimen is reduced [30]. 

In polymorphic PTLD (P-PTLD) the underlying lymphoid structure is effaced by an extensive proliferation of stromal immune cells (plasma cells, lymphocytes, histiocytes, and eosinophils) and few transformed cells, similarly to classical Hodgkin lymphoma. Although clonal genomic alterations are rare in P-PTLD, *BCL-6* is mutated in 50% of P-PTLD and associated with aggressive disease [31]. Most P-PTLD that arise within one year following transplantation are monoclonal and EBV-positive [32, 33].

Monomorphic PTLD (M-PTLD) represents a group of neoplastic lymphoproliferations corresponding to similar pathologic entities in immunocompetent individuals [17]. More than 80% of the cases are NHL of which mainly DLBCL although sporadic cases of post-transplant Burkitt's lymphoma, plasmablastic lymphoma, and plasma cell myeloma/Kahler's disease have also been documented. T-cell or natural killer (NK) cell are only rarely encountered in the western world [2, 18]. Posttransplant Hodgkin lymphoma, although rare, is usually regarded as a fourth distinct category [30]. The majority of M-PTLD contains the EBV genome and clonal rearrangements of immunoglobulin genes [34].

E-PTLD and P-PTLD lesions generally arise earlier following transplantation than M-PTLD. A possible explanation is that E- and P-PTLD develop due to an aberrant response to EBV that may be introduced via the graft. This also explains why the vast majority of E- and P-PTLD are EBV-positive although exact percentages vary. One study detected EBV-positive lymphocytes in only 67% of FFH [35]. This observation suggests that EBV-positive and -negative early posttransplant lymphoproliferative lesions can present with similar morphological characteristics. 

The onset of P-PTLD and M-PTLD may overlap but is variable, partly because diagnosis depends on when these lesions become clinically detectable. Because M-PTLD is thought to arise from E- and P-PTLD, the majority of M-PTLD is EBV-positive. Within the M-PTLD group, EBV-positive lymphomas arise earlier following transplantation than EBV-negative lymphomas [36]. This observation supports the hypothesis that EBV accelerates (malignant) transformation of B-cells and promotes aberrant B-cell proliferation. 

## 4. The Epstein-Barr Virus (EBV) Exploits B-Cell Differentiation Pathways

### 4.1. EBV Can Persist Latently in Infected B-Cells

EBV is the first virus that has been associated with oncogenesis and is one of the most effectively transforming viruses *in vitro*. In contrast, it was shown that EBV infection *in vivo *induces transient proliferation and latent persistence in nonpathogenic memory B-cells rather than (immediate) transformation. This explains why EBV can persist benignly for a lifetime in over 90% of human hosts and also why not every infected individual develops cancer, even when immunocompromised. Additional aberrations in B-cell biology or viral infective mechanisms are required to prevent latent infection from being established and allow cancer to develop [37]. 

Upon B-cell infection, the linear viral genome circularizes and is maintained as a nuclear episome that can integrate into the host genome [38]. The life cycle of EBV is characterized by an alteration between the lytic phase and a longer period of latency, in which three different latency expression profiles are recognized, each characterized by a specific expression pattern of 9 viral proteins: 6 EBV nuclear antigens (EBNA 1, 2, 3A–C and EBNA-leader protein) and 3 latent membrane proteins (LMP 1, 2A-B) [39]. These latency types are coordinately expressed during differentiation of the infected B-cell and each is associated with specific pathologies [39]. Newly infected B-cells express latency III (or the “growth program”) triggering the expression of all 9 viral proteins. Latency II (also called the “default program,” characterized by LMP1+/EBNA2−) is expressed in cells entering the germinal center reaction that mediates antibody affinity maturation whereas memory cells express latency I (or the “latency program,” characterized by LMP1−/EBNA2−) [40]. Although PTLD has been associated with latency III expression, this profile is probably only required in the early phases of B-cell infection as viral gene expression may vary within and between tumors [41].

The coordinated expression of viral proteins has been associated with specific methylation patterns at their promoter sites [42].

### 4.2. Different Viral Gene Products Contribute to Lymphomagenesis

Upon EBV infection, the first viral protein to be expressed is EBNA2, a potent inducer of viral (LMP1 and LMP2A) as well as cellular proteins (*C-MYC*, IL-18 receptor, etc.) [43]. LMP1 and LMP2A are 2 major viral oncoproteins mimicking cellular CD40, a transmembrane costimulatory protein required for activation of antigen-presenting cells, and a constitutively active B-cell receptor (BCR), respectively. LMP1 can activate NFKB signaling, AKT, and mitogen-activated proteins kinases (MAPK: p38, ERK, JNK involved in the growth and survival of transformed cells) and plays an important role in the survival of infected B-cells going through the germinal center reaction [3, 38]. Furthermore, LMP1 modulates several genes involved in apoptosis (*c-FLIP*, an apoptosis inhibitor, *BCL-2*) and cytokine expression. It upregulates *IL-10* that functions as an autocrine B-cell growth factor, and via induction of *IFN-gamma*, LMP1 indirectly upregulates STAT1 resulting in a sustained expression of CXCL9 and p21 [44].

LMP2A is localized in the B-cell membrane and binds tyrosine kinases eventually impairing BCR-mediated activation and entry of the lytic cycle. To ensure survival of the infected cells LMP2A can induce the vital signals normally provided by BCR signaling [45]. 

Together, LMP1 and 2A stimulate the infected B-cell to become a proliferating blast and guide it throughout the germinal center reaction ultimately driving the infected lymphocyte towards the memory cell stage where EBV can persist. At this stage, viral expression is shut down minimizing immunogenicity of the infected cells. Only during memory cell division, EBNA1 is expressed to ensure replication of the viral episome. When the memory cell differentiates into a plasma cell in response to antigen stimulation, EBV enters the lytic cycle producing new infectious particles that are shed in the saliva. From this infectious mechanism, it becomes clear that the oncogenic potential of EBV becomes substantial when the B-cell lymphoblast is unable to differentiate or when the growth program is aberrantly expressed in the absence of an effective cytotoxic T-lymphocytes (CTLs) response. Under normal conditions CTLs recognize and kill EBV-infected lymphoblasts, but in case of immunodeficiency (like posttransplantation), infected cells can proliferate uncontrollably. Nevertheless, this is still a rare event as only 1 or 2 of millions of EBV-infected cells eventually develop into tumors [46]. 

Other gene products encoded by the EBV genome include EBV-encoded RNAs (EBERs), miRNAs (19- to 25-nucleotide-long single-stranded RNAs, [47]), and a series of proteins that are homologous to or interact with cellular antiapoptotic proteins, signal transducers and cytokines (e.g., viral *IL-10* and *BCL-2*) and mediate the pathogenic, and oncogenic effects of EBV [48, 49]. 

The 2 EBERs are expressed in all latency programs but their function remains unclear although they represent the most abundantly expressed viral products in most infected cells [50]. It is assumed that in Burkitt's lymphoma they inhibit apoptosis induced by *C-MYC *translocation and induce *IL-10* expression resulting in B-cell survival and immunosuppression [51, 52]. 

In the EBV genome, miRNAs expressed during latency are encoded in *BART* clusters (in the introns of the *BART* gene) and *BHRF-1* clusters (in the 3′ UTR of the *BHRF-1* open reading frame) and can regulate cellular genes, possibly conferring resistance to apoptosis [53]. Interestingly, the *BHRF-1*-encoded protein is the viral homolog of *BCL-2*. Furthermore, EBV miRNAs can downregulate viral proteins such as LMP1 and 2A representing a possible mechanism for immune escape (reviewed in [54]). Studies have shown that EBV-encoded miRNAs are shed via exosomes, possibly interfering with the immune response against EBV-infected cells [55]. Expression of viral miRNAs however is very complex and has been shown to be tissue specific and dependent on the pattern of the EBV gene expression [50]. Aside from its own miRNAs, EBV can simultaneously induce cellular miRNAs for the modulation of interferon responses (miR-146a, [56]) and lymphocyte homeostasis (miR-155, [57]).

## 5. Genetic Studies Gradually Unravel the Molecular Basis of Posttransplant B-Cell Lymphoma

So far, no genetic aberrations have been associated exclusively with EBV-positive or -negative PTLD suggesting that EBV infection alone is not sufficient for lymphomagenesis but that (coincidental) preceding and/or subsequent genetic events are required for full transformation of a B-cell. However, increased expression of BCL-6, decreased expression of MUM1 (multiple myeloma oncogene 1), and a more frequent germinal center-derived cell of origin have been associated with EBV-negative PTLD [33]. Furthermore, a number of studies have shown that the transcriptional profiles of EBV-positive and -negative PTLD differ substantially, illustrating the impact of EBV infection on cell signaling. 

### 5.1. Gene Expression Profiling

Genomic profiling is warranted to shed light on the molecular pathogenesis of PTLD, but the 2 small gene expression profiling (GEP) studies that have been performed on PTLD yielded somewhat conflicting results. 

Craig et al. performed a microarray analysis on a group of 4 EBV-positive and 4 EBV-negative M-PTLD patients and demonstrated segregation of PTLD cases based on the EBV status. Antiviral immune responses and cell cycle proteins were upregulated in EBV-positive PTLD, whereas components of the BCR and their downstream signaling on the other hand were downregulated [58]. The group of Vakiani et al. could not confirm the segregation of EBV-positive and -negative posttransplant tumors but showed that PTLD were clearly distinct from immunocompetent NHL, based on a study including 12 PTLD patients [59]. 

Clearly, no consensus exists on the role of EBV and the molecular features of PTLD. Based on a microarray experiment performed by our lab and comprising 48 DLBCL cases of which 33 occurred following transplantation (72% EBV-positive) and 15 in immunocompetent hosts (none EBV-positive), we concluded that EBV-positive and EBV-negative PTLD are characterized by a distinct gene expression profile. Innate and tolerogenic immune responses played a central role in EBV-positive posttransplant DLBCL as opposed to EBV-negative posttransplant DLBCL. In addition, except for decreased T-cell signaling, these latter cases coincided with EBV-negative DLBCL occurring in immunocompetent individuals suggesting that EBV-negative posttransplant lymphomas are coincidental cases of lymphoma biologically similar to lymphoma in the immunocompetent population [6]. 

### 5.2. Conventional Cytogenetics and Comparative Genomic Hybridization

Multiple genetic alterations that have been associated with PTLD (Table 1) suggest that EBV infection alone does not account for posttransplant lymphomagenesis. Different mechanisms have already been identified that contribute to this process. Cytogenetic analysis of 36 PTLD has demonstrated that 72% of monomorphic B-cell PTLD contain chromosomal abnormalities, as opposed to 15% of polymorphic PTLD and none of the early lesions. The most frequent aberrations were trisomies of chromosome 9 and/or 11 associated with EBV positivity, followed by translocations involving 8q24.1 (*C-MYC*), 3q27 (*BCL-6*), and 14q32 (*IGH, TCL1*) [60]. Two other studies reported different frequencies of cytogenetic abnormalities in PTLD (33% of P-PTLD, 75% of M-PTLD [35], 57% of P-PTLD, and 46% of M-PTLD [21]). These differences suggest that part of PTLD cases are caused by mutations, epigenetic alterations, and oncogenic EBV signaling [61–63].

Comparative genomic hybridization analysis has confirmed the occurrence of these alterations and has shown that PTLD is characterized by distinct genetic aberrations (losses of 4q, 17q, and Xp) as well as changes that are also common in lymphoma arising in immunocompetent patients but with different frequencies (losses of 1p, 6q, 9p, 17p13: *TP53*; gains of 3q27: *BLC-6*, 5p, 7q, 8q24: *C-MYC*, 11p, 12q, 12p, 18q21: *BCL-2 *and *MALT*, and 21q) [21]. Whole genome comparative genomic hybridization on posttransplant DLBCL revealed common gains of 5p and 11p together with losses of 6q, 1p and 9p. In this study, 12p was the most common target of deletion followed by 4p, 4q, 12q, 17p, and 18q [64]. Rinaldi et al. noticed a lack of genetic lesions characteristic for (post)germinal center lymphoma such as gains of chromosome 3 (*FOXP1*, *BCL-6*, and *NFKBIZ*) and 18q (*BCL-2* and *NFATC1*) together with losses of 6q (*PRDM1* and *TNFAIP3*) in posttransplant DLBCL despite the postgerminal origin of most of their reported cases [65]. Furthermore, PTLD would consist of less complex karyotypic aberrations compared to DLBCL in an immunocompetent population [66]. These findings underscore the role of the immunological background in genetic variability of lymphoma.

Comparative high-density genome-wide analysis identified del(2p16.1) targeting the fragile site *FRA2E* as the most common lesion in posttransplant DLBCL [65]. Interestingly, *FRA2E* is very similar to an EBV insertion site discovered in a Burkitt's lymphoma cell line suggesting that the genomic instability of PTLD might be due to integration of viral DNA upon infection. *FRA2E* contains *FANCL* (an ubiquitin ligase important in DNA repair) and *VRK2* (a negative regulator of the MAPK pathway) which may play a role in oncogenesis [65, 67]. 

Comparison of EBV-positive and EBV-negative post-transplant DLBCL showed that the latter contain more recurrent genomic lesions among which del(4q25-q35), gains of 7p, 7q and 11q24-q25. It has been hypothesized that in EBV-negative PTLD (or after hit-and-run infection), genetic alterations accumulate in order to substitute for the oncogenic effects of EBV [65].

Loss of heterozygosity (LOH, i.e., inactivation of an allele of a gene in which the other allele is already dysfunctional) occurs commonly in cancer [68–70]. Interestingly, one study showed that LOH without loss of copy number is prevalent in PTLD especially at 1q, 9p, 10q, and 11q [64]. Theoretically, this could be caused by loss of one allele and subsequent duplication of the remaining one, but this seems rather unlikely. Uniparental disomy (UD), also known as copy-neutral LOH, provides another explanation that also has been reported in other malignancies and results in biallelic inactivation without loss of DNA [71–73]. During mitosis, missegregation of 2 mutated chromosomes gives rise to daughter cells with 3 copies of the same chromosome. When by coincidence the normal chromosome is deleted in an attempt to restore ploidy, the daughter cell eventually possesses 2 mutated chromosomes. Interestingly, UD of the MHCII locus at 6p has not been reported in posttransplant DLBCL in contrast to DLBCL in immunocompetent patients. Decreased expression or absence of MHCII results in reduced infiltration of T-cells and even impaired activation of CTLs contributing to immune escape [74]. It has been hypothesized that due to iatrogenic immunodeficiency posttransplantation, downregulation of MHCII is superfluous [65]. 

In general, posttransplant lymphoma demonstrates a lower frequency of unbalanced genomic aberrations than DLBCL in immunocompetent hosts. This can be explained by the mutator phenotype that is a unique feature of a number of immunosuppression-related lymphomas and which may result in microsatellite instability. The mutator phenotype is induced when loss of a gene, for example, involved in DNA repair, accelerates the accumulation of mutations in numerous other genes with potentially deleterious consequences [3, 75]. 

It is unclear whether different grafts are linked to particular genetic alterations but, remarkably, DLBCL following heart transplantation has been associated with a high prevalence of 6p gains [65].

### 5.3. DNA Sequencing

Nucleotide-level variations can be caused by aberrant somatic hypermutation (SHM) during the germinal center reaction. SHM normally targets the immunoglobulin variable (*IgV*) genes in an attempt to generate high-affinity antibodies [76]. Aberrant SHM is thought to be a tumor-specific pathogenetic process targeting proto-oncogenes such as *PIM-1*, *PAX-5*, *C-MYC*, and *RhoH/TTF* and has been reported in lymphoma to be independent of the immune and EBV status of the host [22, 77] (Table 1). Aberrant SHM may also introduce stop codons in *Ig* genes resulting in crippled BCR. In these cells, LMP2A may function as a BCR substitute providing the necessary survival signals [78].

### 5.4. Polymorphism Analysis

Despite the ubiquity of EBV, cancer driven by EBV is still a relatively rare phenomenon, even in (partially) immunocompromised individuals. Part of the explanation probably lies in the numerous (single-nucleotide) polymorphisms present in the human as well as the viral genome that may affect disease progression at the level of the immune responses and behavior of EBV (Table 1). Recent studies have demonstrated the importance of single nucleotide polymorphisms (SNPs) in cytokines that can influence the outcome of EBV infection in transplant patients [79, 80]. Although a chronically high EBV viral load constitutes a major risk factor for the development of EBV-related lymphoproliferative disorders, not all patients with high EBV serum levels develop symptomatic disease [11]. *IL-1RN* (interleukin 1 receptor antagonist) and *IL-1*β** alleles producing more severe inflammatory responses were found to protect patients against EBV viremia [79]. Analogously, a polymorphism in antiviral *IFN-gamma* that results in decreased *IFN-gamma* synthesis has been associated with early-onset and pediatric PTLD [80, 81]. Furthermore, increased plasma TNF-alpha levels have been detected in EBV-positive PTLD patients [82]. A polymorphism in the *TNF-alpha* promoter has been described to affect transcriptional regulation by NF-KB, potentially resulting in higher TNF-alpha levels [83] and TNF-alpha-induced DNA damage and antiapoptosis. [82, 84]. Interestingly, the same TNF promoter polymorphism has been associated with NHL development in the general population [85]. 

*IL-10* and *TGF-beta* have protumoral (immunosuppressive) as well as antitumoral characteristics (*TGF-beta* blocks B-cell activation and proliferation; *IL-10* enhances antibody responses), and low expression of both has been linked to late-onset EBV-positive PTLD [86]. Altogether, the observation that expression of *IFN-gamma*, *IL-10*, and *TGF-beta* is decreased in PTLD suggests that a shift of the T-helper 1/T-helper 2 (Th1/Th2) balance towards the Th2 pathway plays a role in PTLD development. 

The human leukocyte antigen (*HLA*) system comprises 2 classes of antigen-presenting proteins of which polymorphic variants have been associated with virus-associated cancers [87, 88] and also PTLD development [89, 90]. Potential explanations for the association between *HLA* and PTLD involve interactions of natural killer cells and CTLs with *HLA* proteins. It is also possible that inefficient antigen presentation of EBV proteins contributes to decreased immunoreactivity towards EBV. One study showed that the *HLA-A26* variant conferred a threefold increased risk for PTLD development if it was present in the transplant recipient or donor. It was hypothesized that the latter represents donor-derived PTLD, the prevalence of which might currently be underestimated. The same study also identified a protective *HLA* haplotype linked with a “hyperactive immune system” suggesting that carriers of this haplotype have better immunological defenses against EBV [90]. However, most evidence is derived from small patient populations with isolated but not confirmed findings supporting the contribution of specific donor/patient *HLA* alleles, *HLA* haplotypes, *HLA* mismatches, and preexisting *HLA* antibodies in the development of PTLD in transplant recipients. Clearly, larger studies are needed to further clarify this complex association.

Although the role of innate immunity in PTLD is not fully understood, one study identified a polymorphism in an Fc receptor expressed on natural killer cells that was associated with increased affinity for IgG antibodies resulting in more efficient antibody-mediated cytotoxicity. Carriers of this Fc variant had a significantly improved outcome compared to other PTLD patients [91]. Together, these studies suggest that (genetically predisposed) decreased control of EBV infection can contribute to lymphomagenesis possibly due to increased susceptibility to (iatrogenic) immunosuppression. Because the prognosis of PTLD patients is highly dependent on early diagnosis, the identification of polymorphisms that can function as predictive biomarkers can help define patients at risk and can greatly improve disease outcome. 

At the level of the viral genome, variations that may influence viral load and PTLD development have also been described. The 30-bp deleted LMP1 variant has been associated with lower EBV serum levels compared to wild-type LMP1 [92]. Other studies have revealed differences in signaling properties of LMP1 variants that influence B-cell survival and proliferation [93]. Based on polymorphisms in EBNA proteins and EBER, 2 EBV strains are distinguished (A-type and B-type EBV). Both strains have been detected in immunocompetent as well as immunocompromised individuals [94, 95] and have been shown to differ in their potential to enter the lytic cycle [96–98]; in particular, type B is associated with lytic replication. BZLF1, a protein encoded by the EBV Bam*HI fragment Z*, is a central regulator of the switch from latency to lytic replication [99]. Gutiérrez et al. associated distinct differences within its promoter region with types A and B EBV, respectively. Furthermore, they identified a BZLF1 promoter variant that was exclusively present in EBV-driven nonmalignant lesions [100]. It is possible that EBV encoding this variant has increased ability to lyse the cell in response to physiological stimuli (*TGF-beta*, activation of the B-cell receptor) which would decrease the chance of malignant transformation [96]. This hypothesis is illustrated by a study of Ibrahim et al. who identified type A EBV in the vast majority of their B-cell PTLD series. Type B EBV on the other hand was more prevalent in EBV-positive lymphoma patients with prolonged HIV-associated immunodeficiency suggesting that the type and degree of immunodeficiency are associated with the EBV genotype [101].

### 5.5. Epigenetics

Apart from genetic, also epigenetic alterations and more specifically DNA hypermethylation have been implicated in the pathogenesis of PTLD. DNA methylation is carried out by DNA methyltransferases (DNMT1, 3A-B) counteracted by DNA methylases as a mechanism to fine-tune gene expression. Excess methylation of tumor suppressor genes results in significant downregulation of gene expression and may contribute to cancer development. *DAPK1* (proapoptotic), *MGMT* (involved in gene repair) and *SHP1* (antiproliferative) are a few examples of tumor suppressor genes that are hypermethylated in the majority of M-PTLD [15, 62, 102] (Table 1).

EBV is known to modulate DNA methylation in germinal center B-cells via downregulation of DNMT1 (by LMP1) and DNMT3B and upregulation of DNMT3A resulting in clustered changes in methylation status of cellular genes depending on the CpG content of the promoter region. It has been hypothesized that DNMT3A silences the viral Wp promoter by methylation inducing a switch to the viral Cp promoter resulting in expression of more viral proteins. These observations indicate that EBV infection alters methylation of the host as well as its own genome [103]. Importantly, DNMT1 has been implicated in normal B-cell differentiation and DNA repair suggesting that deregulated expression of DNMTs as a result of EBV infection contributes to lymphomagenesis [104]. 

### 5.6. Proteomics

Ideally, data regarding gene expression and genetic alterations are correlated with evidence at protein level, but proteomic analysis of PTLD is very rarely reported. A study comprising 6 monomorphic PTLD of which 5 were EBV-positive demonstrated upregulation of NFKB, PI3K, Akt, mTOR, MAPK and PKC pathways, cell cycle regulation, endoplasmic reticulum homeostasis (HSP90), and apoptosis-related proteins (caspase 7-8 and MAP2K4). Furthermore, *in vitro* EBV-positive lymphoma was more sensitive to inhibitors of PI3K/mTOR and HPS90 than EBV-negative lymphoma [105]. The differential activity of these inhibitors can be explained by induction of the NFKB pathway (by LMP1), the PI3K/Akt/mTOR pathways (by LMP2A) and heat-shock proteins in EBV-positive PTLD [105]. A complementary immunohistochemical study showed that mTOR is expressed in PTLD independently of the EBV status [106]. It is possible however that EBV-positive PTLD relies more on mTOR signaling than EBV-negative PTLD. Also apoptotic pathways seem to differ between EBV-positive and -negative PTLD: proapoptotic Bim, a critical regulator of lymphocyte survival [107], and apoptosis effector cleaved PARP were shown to be downregulated in EBV-associated PTLD [108].

### 5.7. Microenvironment

The tumor microenvironment refers to the local niche in which tumor cells reside and consists of stromal as well as inflammatory cells. Recent studies have highlighted its importance in oncogenesis, tumor progression, and prognosis, and during the past years, the role of the microenvironment in tumor development has gained significant importance [109, 110]. Depending on the lymphoma subtype and where the tumor arises, the microenvironment can differ substantially [111–113]. Although the immune responses in posttransplant patients are profoundly altered due to the chronically administered immunosuppressive regimen, local infiltration of antitumor cytotoxic T-lymphocytes (CTL) has been observed in PTLD patients and was associated with favorable overall survival [114]. It has been hypothesized that in case of EBV-positive PTLD, CTLs react to viral antigen. However one study observed a high ratio of CD4+/CD8+ T-cells in EBV-positive PTLD suggesting that T-cell infiltrates reflect a general response to immunosuppression rather than to EBV [115]. Most likely, both scenarios are true.

Regulatory T-cell (Treg) infiltration is thought to be consistently restricted in PTLD limiting immunosuppressive effects but also suppression of B-cell proliferation which could potentially contribute to PTLD development [114]. This was confirmed by a study showing reduced Treg cell numbers in liver transplant patients treated with combined immunosuppressive therapy (prednisone/azathioprine/tacrolimus) [116]. 

EBV interacts intensely with the innate immune system (reviewed in [117]) suggesting that EBV-positive and -negative PTLD differ in terms of immunoreactivity. Nevertheless, not much is known about the role of innate immunity in PTLD although a number of interesting observations have been reported. Dendritic cells (DCs) represent a type of antigen-presenting cells comprising 2 principle subsets: myeloid and plasmacytoid DC. Marked infiltration of the latter has been observed in early PTLD lesions in contrast to monomorphic PTLD [118]. Interestingly, EBV-infected cells release exosomes containing viral miRNAs which target different cell types depending of the origin of the exosomes: B-cell-derived exosomes mainly target other B-cells, whereas exosomes secreted from DC are engulfed by monocytes [119].

Only few data are available concerning the cytokine profile of the PTLD microenvironment. *IL-10* is an anti-inflammatory cytokine and a B-cell growth factor. Elevated serum concentration of human *IL-10* has been put forward as a marker for detection of early PTLD development [120, 121] and a combination of 2 SNPs in its promoter region was associated with increased risk for development of B-cell lymphoma [122]. One study showed that at least part of all PTLD cases exhibit a Th2 profile (*IFN-gamma*/IL-2 negative; IL-4/*IL-10* positive; [123]). However, this cytokine profile could be (partially) induced by immunosuppressive therapy following transplantation which has been shown to skew the Th1/Th2 balance in favor of a Th2 response [124] potentially promoting graft acceptance. These insights underscore the delicate immunological balance that when perturbed stimulates either graft rejection (Th1) or tumor growth (Th2).

Matters are further complicated by expression of the viral *IL-10* analogue encoded by *BCRF-1*. Apart from B-cells, EBV can also infect monocytes and macrophages, one of the first cell types to arrive at the site of the viral infection [125, 126]. Because viral *IL-10* is expressed earlier than human *IL-10* following infection, it can efficiently prevent *IFN-gamma*-induced upregulation of primary (MHC-antigen complex) as well as costimulatory (B7, ICAM) signals in myeloid cells eventually impairing antiviral T-cell activation and inducing anergy [127, 128].

Like *IL-10*, IL-6 is a B-cell growth factor of which serum concentration is increased at diagnosis of PTLD. Possibly, IL-6 is expressed by the tumoral cells as part of an autocrine feedback loop providing an explanation for the therapeutic success of an IL-6 antibody [129, 130].

### 5.8. Future Directions for Novel PTLD Therapies

The current options for treatment of B-cell PTLD are largely limited to chemotherapeutic regimens (cyclophosphamide, hydroxyldaunorubicin, oncovin and prednisone, or CHOP therapy) and immunotherapy with Rituximab, an anti-CD20 antibody (as monotherapy or combination therapy with CHOP) [16]. However, because of their immunosuppressive properties, chemotherapeutics are associated with treatment toxicity in posttransplant lymphoma patients more than in immunocompetent patients. For this reason and because overall survival of PTLD patients remains poor, there is a great need for new targeted therapies that efficiently kill tumor cells and decrease the EBV viral load without increasing the risk for graft rejection. Over the years, a number of new therapeutic approaches have been proposed but currently none are routinely used in the clinic. 

Because most PTLD cases are EBV-related, different strategies have been developed to decrease the EBV viral load. Nucleoside inhibitors that inhibit viral replication have little effect due to limited lytic replication of EBV in PTLD [131]. However, preemptive antiviral therapy following transplantation has been associated with decreased risk of PTLD development. Furthermore, when antiviral treatment was preceded by administration of arginine butyrate, an inducer of the EBV lytic cycle, an overall response of 83% was reached in a series of patients with refractory EBV-positive lymphoid malignancies [132]. Promising results have also been reported for infusion of recipient- or donor-derived EBV-specific CTLs but this approach is limited by the labor-intensive procedure and availability problems [133]. To overcome this problem, engineered T-cell receptors (TCRs) consisting of anti-EBV antibody fragments linked to the TCR signaling component have been developed and have shown promising results in treating both autoimmune disorders and malignancy [134, 135]. However, the efficacy of these immunotherapies depends on the expression of viral proteins which varies for different latency types and different tumors [135, 136]. 

Other types of immunotherapy, namely, cytokine therapy and antibody therapy have also been developed. However, administration of antiviral/antitumoral IFN-alpha was poorly tolerated and associated with graft rejection [137], whereas anti-IL-6 antibody therapy showed promising results [129] but is not (yet) widely used.

In recent years, increasing molecular insight in PTLD pathogenesis has been translated in new potential therapeutics. Inhibitors of mTOR have been studied in small clinical trials but their efficacy is debated [138–140]. *In vitro* studies have shown that activation of apoptosis by small molecules is effective against EBV-associated lymphoma which could represent a novel way to treat PTLD [141]. An EBV vaccine is currently in clinical development for use in nasopharyngeal carcinoma patients [142] and may represent the ultimate way to prevent PTLD development.

## 6. Conclusion

Although PTLD pathogenesis is still not well understood, current knowledge of disease development at the DNA, RNA, and protein level underlines its complex etiology. It is important to keep in mind that malignant PTLD comprises many different lymphoma subtypes (DLBCL, Burkitt's lymphoma, plasmablastic lymphoma etc.) of which the disease mechanisms can still differ considerably warranting the development of patient-specific therapies. 

## Figures and Tables

**Table 1:** Most common genetic and epigenetic alterations detected in B-cell posttransplant lymphoma.

Translocations involving	Copy number variations		DNA mutations	DNA polymorphisms	Epigenetic alterations
	**Losses **	**Gains**		**Host genome**	
8q24 (*C-MYC*) 3q27 (*BCL-6*) 14q32 (*IgH, TCL1*)	1p	2p24-p25 (*CD138*)	*PIM1* *PAX5* *C-MYC* *RhoH/TTF *	*IL-10* *TGF-beta* *IFN-gamma* *TNF-alpha* *HLA*	*DAP-k* *MGMT* *SHP1* *TP73 *
	1q (LOH)	3q27 (*BCL-6*)			
	2p16.1 (*FRA2E: FANCL, VRK2*)	4q21.21 5p			
	6q (*PRDM1, TNFAIP3*)	7q 8q24 (*C-MYC*)		**EBV genome** *LMP1* *BZLF1 *	
	9p (LOH)	9 (EBV+)			
	10q (LOH)	9p			
	11q (LOH)	11 (EBV+)			
	12	11p			
	17p13 (*TP53*)	12p			
	17p	12q			
	18q	18q21 (*BCL-2, MALT1*)			
	4q (rare)				
	Xp (rare)	21q			

Abbreviations: EBV: Epstein-Barr Virus; FRA: fragile site; LOH: loss of heterozygosity.

## References

[1] Dierickx D, Tousseyn T, Sagaert X (2013). Single-center analysis of biopsy-confirmed posttransplant lymphoproliferative disorder: incidence, clinico-pathological characteristics and prognostic factors. *Leukemia and Lymphoma*.

[2] Herreman A, Dierickx D, Morscio J (2013). Clinicopathological characteristics of Posttransplant Lymphoproliferative Disorders of T-cell origin: single center series of 9 cases and meta-analysis of 147 reported cases. *Leukemia and Lymphoma*.

[3] Capello D, Gaidano G (2009). Post-transplant lymphoproliferative disorders: role of viral infection, genetic lesions and antigen stimulation in the pathogenesis of the disease. *Mediterranean Journal of Hematology and Infectious Diseases*.

[4] Manez R, Breinig MC, Linden P (1997). Posttransplant lymphoproliferative disease in primary Epstein-Barr virus infection after liver transplantation: the role of cytomegalovirus disease. *Journal of Infectious Diseases*.

[5] Jox A, Rohen C, Belge G (1997). Integration of Epstein-Barr virus in Burkitt’s lymphoma cells leads to a region of enhanced chromosome instability. *Annals of Oncology*.

[6] Morscio J, Dierickx D, Ferreiro JF, Herreman A, van Loo P, Bittoun E Gene expression profiling reveals clear differences between EBV-positive and EBV-negative post-transplant lymphoproliferative disorders.

[7] Opelz G, Henderson R (1993). Incidence of non-Hodgkin lymphoma in kidney and heart transplant recipients. *The Lancet*.

[8] Alizadeh AA, Elsen MB, Davis RE (2000). Distinct types of diffuse large B-cell lymphoma identified by gene expression profiling. *Nature*.

[9] Capello D, Rossi D, Gaidano G (2005). Post-transplant lymphoproliferative disorders: molecular basis of disease histogenesis and pathogenesis. *Hematological Oncology*.

[10] Johnson LR, Nalesnik MA, Swerdlow SH (2006). Impact of Epstein-Barr virus in monomorphic B-cell posttransplant lymphoproliferative disorders: a histogenetic study. *American Journal of Surgical Pathology*.

[11] Green M, Soltys K, Rowe DT, Webber SA, Mazareigos G (2009). Chronic high Epstein-Barr viral load carriage in pediatric liver transplant recipients. *Pediatric Transplantation*.

[12] Schaffer K, Hassan J, Staines A (2011). Surveillance of Epstein-Barr virus loads in adult liver transplantation: associations with age, sex, posttransplant times, and transplant indications. *Liver Transplantation*.

[13] Shapiro RS, McCLain K, Frizzera G (1988). Epstein-Barr virus associated B cell lymphoproliferative disorders following bone marrow transplantation. *Blood*.

[14] Dharnidharka VR, Dharnidharka VR, Green M, Webber SA (2010). Epidemiology of PTLD. *Post-Transplant Lymphoproliferative Disorders*.

[15] Ibrahim HA, Naresh KN (2012). Posttransplant lymphoproliferative disorders. *Advances in Hematology*.

[16] Murukesan V, Mukherjee S (2012). Managing post-transplant lymphoproliferative disorders in solid-organ transplant recipients: a review of immunosuppressant regimens. *Drugs*.

[17] Nourse JP, Jones K, Gandhi MK (2011). Epstein-Barr virus-related post-transplant lymphoproliferative disorders: pathogenetic insights for targeted therapy. *American Journal of Transplantation*.

[18] LaCasce AS (2006). Post-transplant lymphoproliferative disorders. *Oncologist*.

[19] Curtis RE, Travis LB, Rowlings PA (1999). Risk of lymphoproliferative disorders after bone marrow transplantation: a multi-institutional study. *Blood*.

[20] Lucas KG, Small TN, Heller G, Dupont B, O’Reilly RJ (1996). The development of cellular immunity to Epstein-Barr virus after allogeneic bone marrow transplantation. *Blood*.

[21] Poirel HA, Bernheim A, Schneider A (2005). Characteristic pattern of chromosomal imbalances in posttransplantation lymphoproliferative disorders: correlation with histopathological subcategories and EBV status. *Transplantation*.

[22] Capello D, Rasi S, Oreste P (2009). Molecular characterization of post-transplant lymphoproliferative disorders of donor origin occurring in liver transplant recipients. *The Journal of Pathology*.

[23] Weissmann DJ, Ferry JA, Harris NL, Louis DN, Delmonico F, Spiro I (1995). Posttransplantation lymphoproliferative disorders in solid organ recipients are predominantly aggressive tumors of host origin. *American Journal of Clinical Pathology*.

[24] Quinlan SC, Pfeiffer RM, Morton LM, Engels EA (2011). Risk factors for early-onset and late-onset post-transplant lymphoproliferative disorder in kidney recipients in the United States. *American Journal of Hematology*.

[25] Leblond V, Dhedin N, Bruneel MFM (2001). Identification of prognostic factors in 61 patients with posttransplantation lymphoproliferative disorders. *Journal of Clinical Oncology*.

[26] Jain A, Nalesnik M, Reyes J (2002). Posttransplant lymphoproliferative disorders in liver transplantation: a 20-year experience. *Annals of Surgery*.

[27] Mucha K, Foroncewicz B, Ziarkiewicz-Wróblewska B, Krawczyk M, Lerut J, Pczek L (2010). Post-transplant lymphoproliferative disorder in view of the new WHO classification: a more rational approach to a protean disease?. *Nephrology Dialysis Transplantation*.

[28] Domingo-Domenech E, de Sanjose S, Gonzalez-Barca E (2001). Post-transplant lymphomas: a 20-year epidemiologic, clinical and pathologic study in a single center. *Haematologica*.

[29] Oton A, Wang H, Leleu X (2008). Clinical and pathological prognostic markers for survival in adult patients with post-transplant lymphoproliferative disorders in solid transplant. *Leukemia and Lymphoma*.

[30] Swerdlow SH, Campo E, Harris NL (2008). *WHO Classification of Tumours of Haematopoietic and Lymphoid Tissues*.

[31] Cesarman E, Chadburn A, Liu YF, Migliazza A, Dalla-Favera R, Knowles DM (1998). BCL-6 gene mutations in posttransplantation lymphoproliferative disorders predict response to therapy and clinical outcome. *Blood*.

[32] Shaknovich R, Basso K, Bhagat G (2006). Identification of rare Epstein-Barr virus infected memory B cells and plasma cells in non-monomorphic post-transplant lymphoproliferative disorders and the signature of viral signaling. *Haematologica*.

[33] Jagadeesh D, Woda BA, Draper J, Evens AM (2012). Post transplant lymphoproliferative disorders: risk, classification, and therapeutic recommendations. *Current Treatment Options in Oncology*.

[34] Evens AM, Roy R, Sterrenberg D, Moll MZ, Chadburn A, Gordon LI (2010). Post-transplantation lymphoproliferative disorders: diagnosis, prognosis, and current approaches to therapy. *Current Oncology Reports*.

[35] Vakiani E, Nandula SV, Subramaniyam S (2007). Cytogenetic analysis of B-cell posttransplant lymphoproliferations validates the World Health Organization classification and suggests inclusion of florid follicular hyperplasia as a precursor lesion. *Human Pathology*.

[36] Nalesnik MA (2001). The diverse pathology of post-transplant lymphoproliferative disorders: the importance of a standardized approach. *Transplant Infectious Disease*.

[37] Thorley-Lawson DA (2005). EBV the prototypical human tumor virus—just how bad is it?. *Journal of Allergy and Clinical Immunology*.

[38] Thorley-Lawson DA (2001). Epstein-Barr virus: exploiting the immune system. *Nature Reviews Immunology*.

[39] da Silva SR, de Oliveira DE (2011). HIV, EBV and KSHV: viral cooperation in the pathogenesis of human malignancies. *Cancer Letters*.

[40] Hamilton-Dutoit SJ, Rea D, Raphael M (1993). Epstein-Barr virus-latent gene expression and tumor cell phenotype in acquired immunodeficiency syndrome-related non-Hodgkin’s lymphoma: correlation of lymphoma phenotype with three distinct patterns of viral latency. *American Journal of Pathology*.

[41] Ocheni S, Olusina DB, Oyekunle AA (2010). EBV-associated malignancies. *Open Infectious Diseases Journal*.

[42] Takacs M, Banati F, Koroknai A (2009). Epigenetic regulation of latent Epstein-Barr virus promoters. *Biochimica et Biophysica Acta*.

[43] Pagès F, Galon J, Karaschuk G (2005). Epstein-Barr virus nuclear antigen 2 induces interleukin-18 receptor expression in B cells. *Blood*.

[44] Vaysberg M, Lambert SL, Krams SM, Martinez OM (2009). Activation of the JAK/STAT pathway in epstein barr virus^+^-associated posttransplant lymphoproliferative disease: role of interferon-*γ*. *American Journal of Transplantation*.

[45] Hopwood P, Crawford DH (2000). The role of EBV in post-transplant malignancies: a review. *Journal of Clinical Pathology*.

[46] Thorley-Lawson DA, Gross A (2004). Persistence of the Epstein-Barr virus and the origins of associated lymphomas. *The New England Journal of Medicine*.

[47] Bartel DP (2004). MicroRNAs: genomics, biogenesis, mechanism, and function. *Cell*.

[48] Rezk SA, Weiss LM (2007). Epstein-Barr virus-associated lymphoproliferative disorders. *Human Pathology*.

[49] Thompson MP, Kurzrock R (2004). Epstein-Barr virus and cancer. *Clinical Cancer Research*.

[50] Swaminathan S (2008). Noncoding RNAs produced by oncogenic human herpesviruses. *Journal of Cellular Physiology*.

[51] Samanta M, Iwakiri D, Takada K (2008). Epstein-Barr virus-encoded small RNA induces IL-10 through RIG-I-mediated IRF-3 signaling. *Oncogene*.

[52] Takada K (2001). Role of Epstein-Barr virus in Burkitt’s lymphoma. *Current Topics in Microbiology and Immunology*.

[53] Edwards RH, Marquitz AR, Raab-Traub N (2008). Epstein-Barr virus BART microRNAs are produced from a large intron prior to splicing. *Journal of Virology*.

[54] Barth S, Meister G, Grasser FA (2011). EBV-encoded miRNAs. *Biochimica et Biophysica Acta*.

[55] Zomer A, Vendrig T, Hopmans ES, van Eijndhoven M, Middeldorp JM, Pegtel DM (2010). Exosomes: fit to deliver small RNA. *Communicative & Integrative Biology*.

[56] Cameron JE, Yin Q, Fewell C (2008). Epstein-Barr virus latent membrane protein 1 induces cellular microRNA miR-146a, a modulator of lymphocyte signaling pathways. *Journal of Virology*.

[57] Costinean S, Zanesi N, Pekarsky Y (2006). Pre-B cell proliferation and lymphoblastic leukemia/high-grade lymphoma in E(*μ*)-miR155 transgenic mice. *Proceedings of the National Academy of Sciences of the United States of America*.

[58] Craig FE, Johnson LR, Harvey SAK (2007). Gene expression profiling of epstein-barr virus-positive and -negative monomorphic B-cell posttransplant lymphoproliferative disorders. *Diagnostic Molecular Pathology*.

[59] Vakiani E, Basso K, Klein U (2008). Genetic and phenotypic analysis of B-cell post-transplant lymphoproliferative disorders provides insights into disease biology. *Hematological Oncology*.

[60] Djokic M, Le Beau MM, Swinnen LJ (2006). Post-transplant lymphoproliferative disorder subtypes correlate with different recurring chromosomal abnormalities. *Genes Chromosomes and Cancer*.

[61] Cerri M, Capello D, Muti G (2004). Aberrant somatic hypermutation in post-transplant lymphoproliferative disorders. *British Journal of Haematology*.

[62] Rossi D, Gaidano G, Gloghini A (2003). Frequent aberrant promoter hypermethylation of O^6^-methylguanine-DNA methyltransferase and death-associated protein kinase genes in immunodeficiency-related lymphomas. *British Journal of Haematology*.

[63] Küppers R (2003). B cells under influence: transformation of B cells by Epstein-Barr virus. *Nature Reviews Immunology*.

[64] Rinaldi A, Kwee I, Poretti G (2006). Comparative genome-wide profiling of post-transplant lymphoproliferative disorders and diffuse large B-cell lymphomas. *British Journal of Haematology*.

[65] Rinaldi A, Capello D, Scandurra M (2010). Single nucleotide polymorphism-arrays provide new insights in the pathogenesis of post-transplant diffuse large B-cell lymphoma. *British Journal of Haematology*.

[66] Cigudosa JC, Parsa NZ, Louie DC, Filippa DA, Jhanwar SC, Johansson B (1999). Cytogenetic analysis of 363 consecutively ascertained diffuse large B-cell lymphomas. *Genes, Chromosomes & Cancer*.

[67] Luo WJ, Takakuwa T, Ham MF (2004). Epstein-Barr virus is integrated between REL and BCL-11A in American Burkitt lymphoma cell line (NAB-2). *Laboratory Investigation*.

[68] Adams J, Williams SV, Aveyard JS, Knowles MA (2005). Loss of heterozygosity analysis and DNA copy number measurement on 8 p in bladder cancer reveals two mechanisms of allelic loss. *Cancer Research*.

[69] Idbaih A, Ducray F, Dehais C (2012). SNP array analysis reveals novel genomic abnormalities including copy neutral loss of heterozygosity in anaplastic oligodendrogliomas. *PloS ONE*.

[70] Abkevich V, Timms KM, Hennessy BT (2012). Patterns of genomic loss of heterozygosity predict homologous recombination repair defects in epithelial ovarian cancer. *British Journal of Cancer*.

[71] Matsuse M, Sasaki K, Nishihara E (2012). Copy number alteration and uniparental disomy analysis categorizes Japanese papillary thyroid carcinomas into distinct groups. *PloS ONE*.

[72] Saeki H, Kitao H, Yoshinaga K (2011). Copy-neutral loss of heterozygosity at the p53 locus in carcinogenesis of esophageal squamous cell carcinomas associated with p53 mutations. *Clinical Cancer Research*.

[73] Middeldorp A, van Eijk R, Oosting J (2012). Increased frequency of 20 q gain and copy-neutral loss of heterozygosity in mismatch repair proficient familial colorectal carcinomas. *International Journal of Cancer*.

[74] Booman M, Douwes J, Glas AM (2006). Mechanisms and effects of loss of human leukocyte antigen class II expression in immune-privileged site-associated B-cell lymphoma. *Clinical Cancer Research*.

[75] Duval A, Raphael M, Brennetot C (2004). The mutator pathway is a feature of immunodeficiency-related lymphomas. *Proceedings of the National Academy of Sciences of the United States of America*.

[76] Küppers R (2003). Somatic hypermutation and B cell receptor selection in normal and transformed human B cells. *Annals of the New York Academy of Sciences*.

[77] Pasqualucci L, Neumeister P, Goossens T (2001). Hypermutation of multiple proto-oncogenes in B-cell diffuse large-cell lymphomas. *Nature*.

[78] Dolcetti R (2007). B lymphocytes and Epstein-Barr virus: the lesson of post-transplant lymphoproliferative disorders. *Autoimmunity Reviews*.

[79] Kasztelewicz B, Jankowska I, Pawlowska J, Teisseyre J, Dzierzanowska-Fangrat K (2012). The impact of cytokine gene polymorphisms on Epstein-Barr virus infection outcome in pediatric liver transplant recipients. *Journal of Clinical Virology*.

[80] Lee TC, Savoldo B, Barshes NR (2006). Use of cytokine polymorphisms and Epstein-Barr virus viral load to predict development of post-transplant lymphoproliferative disorder in paediatric liver transplant recipients. *Clinical Transplantation*.

[81] Vanbuskirk AM, Malik V, Xia D, Pelletier RP (2001). A gene polymorphism associated with posttransplant lymphoproliferative disorder. *Transplantation Proceedings*.

[82] McAulay KA, Haque T, Crawford DH (2009). Tumour necrosis factor gene polymorphism: a predictive factor for the development of post-transplant lymphoproliferative disease. *British Journal of Cancer*.

[83] Udalova IA, Richardson A, Denys A (2000). Functional consequences of a polymorphism affecting NF-*κ*B p50-p50 binding to the TNF promoter region. *Molecular and Cellular Biology*.

[84] Reshef R, Luskin MR, Kamoun M (2011). Association of HLA polymorphisms with post-transplant lymphoproliferative disorder in solid-organ transplant recipients. *American Journal of Transplantation*.

[85] Spink CF, Keen LJ, Mensah FK, Law GR, Bidwell JL, Morgan GJ (2006). Association between non-Hodgkin lymphoma and haplotypes in the TNF region. *British Journal of Haematology*.

[86] Babel N, Vergopoulos A, Trappe RU (2007). Evidence for genetic susceptibility towards development of posttransplant lymphoproliferative disorder in solid organ recipients. *Transplantation*.

[87] Hjalgrim H, Rostgaard K, Johnson PCD (2010). HLA-A alleles and infectious mononucleosis suggest a critical role for cytotoxic T-cell response in EBV-related Hodgkin lymphoma. *Proceedings of the National Academy of Sciences of the United States of America*.

[88] Ioannidis JPA, Skolnik PR, Chalmers TC, Lau J (1995). Human leukocyte antigen associations of epidemic Kaposi’s sarcoma. *AIDS*.

[89] Subklewe M, Marquis R, Choquet S (2006). Association of human leukocyte antigen haplotypes with posttransplant lymphoproliferative disease after solid organ transplantation. *Transplantation*.

[90] Pourfarziani V, Einollahi B, Taheri S, Nemati E, Nafar M, Kalantar E (2007). Associations of Human Leukocyte Antigen (HLA) haplotypes with risk of developing lymphoproliferative disorders after renal transplantation. *Annals of Transplantation*.

[91] Stern M, Opelz G, Döhler B, Hess C (2010). Natural killer-cell receptor polymorphisms and posttransplantation non-Hodgkin lymphoma. *Blood*.

[92] Kasztelewicz B, Jankowska I, Pawlowska J, Teisseyre J, Dzierzanowska-Fangrat K (2011). Epstein-Barr virus gene expression and latent membrane protein 1 gene polymorphism in pediatric liver transplant recipients. *Journal of Medical Virology*.

[93] Johnson RJ, Stack M, Hazlewood SA (1998). The 30-base-pair deletion in Chinese variants of the Epstein-Barr virus LMP1 gene is not the major effector of functional differences between variant LMP1 genes in human lymphocytes. *Journal of Virology*.

[94] Srivastava G, Wong KY, Chiang AKS, Lam KY, Tao Q (2000). Coinfection of multiple strains of Epstein-Barr virus in immunocompetent normal individuals: reassessment of the viral carrier state. *Blood*.

[95] Chen HL, Lung ML, Chan KH, Griffin BE, Ng MH (1996). Tissue distribution of Epstein-Barr virus genotypes. *Journal of Virology*.

[96] Buck M, Cross S, Krauer K, Kienzle N, Sculley TB (1999). A-type and B-type Epstein-Barr virus differ in their ability to spontaneously enter the lytic cycle. *Journal of General Virology*.

[97] Lin JC, Lin SC, De BK, Chan WP, Evatt BL (1993). Precision of genotyping of Epstein-Barr virus by polymerase chain reaction using three gene loci (EBNA-2, EBNA-3C, and EBER): predominance of type A virus associated with Hodgkin’s disease. *Blood*.

[98] Sample J, Young L, Martin B (1990). Epstein-Barr virus types 1 and 2 differ in their EBNA-3A, EBNA-3B, and EBNA-3C genes. *Journal of Virology*.

[99] Amon W, Farrell PJ (2005). Reactivation of Epstein-Barr virus from latency. *Reviews in Medical Virology*.

[100] Gutiérrez MI, Ibrahim MM, Dale JK, Greiner TC, Straus SE, Bhatia K (2002). Discrete alterations in the BZLF1 promoter in tumor and non-tumor-associated Epstein-Barr virus. *Journal of the National Cancer Institute*.

[101] Ibrahim HAH, Menasce LP, Pomplun S, Burke M, Bower M, Naresh KN (2010). Epstein-Barr virus (EBV) genotypes among human immunodeficiency virus (HIV)-related B-cell Lymphomas and B-cell post-transplant lymphoproliferative disorders (B-PTLD)-late-onset lymphomas, especially in the HIV setting, are associated with type-B-EBV. *European Journal of Haematology*.

[102] Rossi D, Capello D, Gloghini A (2004). Aberrant promoter methylation of multiple genes throughout the clinico-pathologic spectrum of B-cell neoplasia. *Haematologica*.

[103] Leonard S, Wei W, Anderton J, Vockerodt M, Rowe M, Murray PG (2011). Epigenetic and transcriptional changes which follow Epstein-Barr virus infection of germinal center B cells and their relevance to the pathogenesis of Hodgkin's lymphoma. *Journal of Virology*.

[104] Shaknovich R, Cerchietti L, Tsikitas L (2011). DNA methyltransferase 1 and DNA methylation patterning contribute to germinal center B-cell differentiation. *Blood*.

[105] Alsayed Y, Leleu X, Leontovich A (2008). Proteomics analysis in post-transplant lymphoproliferative disorders. *European Journal of Haematology*.

[106] El-Salem M, Raghunath PN, Marzec M (2007). Constitutive activation of mTOR signaling pathway in post-transplant lymphoproliferative disorders. *Laboratory Investigation*.

[107] Pĩon JD, Labi V, Egle A, Villunger A (2008). Bim and Bmf in tissue homeostasis and malignant disease. *Oncogene*.

[108] Ghigna MR, Reineke T, Rince P (2013). Epstein-barr virus infection and altered control of apoptotic pathways in posttransplant lymphoproliferative disorders. *Pathobiology*.

[109] Badoual C, Hans S, Merillon N (2012). PD-1-expressing tumor-infiltrating T cells are a favorable prognostic biomarker in HPV associated head and neck cancer. *Cancer Research*.

[110] Simpson KD, Templeton DJ, Cross JV (2012). Macrophage migration inhibitory factor promotes tumor growth and metastasis by inducing myeloid-derived suppressor cells in the tumor microenvironment. *Journal of Immunology*.

[111] Kuppers R, Engert A, Hansmann ML (2012). Hodgkin lymphoma. *The Journal of Clinical Investigation*.

[112] Kridel R, Sehn LH, Gascoyne RD (2012). Pathogenesis of follicular lymphoma. *The Journal of Clinical Investigation*.

[113] Zhang L, Yang J, Qian J (2012). Role of the microenvironment in mantle cell lymphoma: IL-6 is an important survival factor for the tumor cells. *Blood*.

[114] Richendollar BG, Tsao RE, Elson P (2009). Predictors of outcome in post-transplant lymphoproliferative disorder: an evaluation of tumor infiltrating lymphocytes in the context of clinical factors. *Leukemia and Lymphoma*.

[115] Perera SM, Thomas JA, Burke M, Crawford DH (1998). Analysis of the T-cell micro-environment in Epstein-Barr virus-related post-transplantation B lymphoproliferative disease. *The Journal of Pathology*.

[116] Verdonk RC, Haagsma EB, Jonker MR (2007). Effects of different immunosuppressive regimens on regulatory T-cells in noninflamed colon of liver transplant recipients. *Inflammatory Bowel Diseases*.

[117] Martorelli D, Muraro E, Merlo A (2012). Exploiting the interplay between innate and adaptive immunity to improve immunotherapeutic strategies for Epstein-Barr-virus-driven disorders. *Clinical and Developmental Immunology*.

[118] Ibrahim HAH, Menasce L, Pomplun S, Burke M, Bower M, Naresh KN (2011). Tumour infiltrating plasmacytoid dendritic cells in B cell post-transplant lymphoproliferative disorders, human immunodeficiency virus-associated B cell lymphomas and immune competent diffuse large B cell lymphomas. *Histopathology*.

[119] Vallhov H, Gutzeit C, Johansson SM (2011). Exosomes containing glycoprotein 350 released by EBV-transformed B cells selectively target B cells through CD21 and block EBV infection in vitro. *Journal of Immunology*.

[120] Birkeland SA, Bendtzen K, Møller B, Hamilton-Dutoit S, Andersen HK (1999). Interleukin-10 and posttransplant lymphoproliferative disorder after kidney transplantation. *Transplantation*.

[121] Muti G, Klersy C, Baldanti F (2003). Epstein-barr virus (EBV) load and interleukin-10 in EBV-positive and EBV-negative post-transplant lymphoproliferative disorders. *British Journal of Haematology*.

[122] Lan Q, Zheng T, Rothman N (2006). Cytokine polymorphisms in the Th1/Th2 pathway and susceptibility to non-Hodgkin lymphoma. *Blood*.

[123] Nalesnik MA, Zeevi A, Randhawa PS (1999). Cytokine mRNA profiles in Epstein-Barr virus-associated post-transplant lymphoproliferative disorders. *Clinical Transplantation*.

[124] Kosmidis M, Dziunycz P, Suarez-Farinas M (2010). Immunosuppression affects CD4+ mRNA expression and induces Th2 dominance in the microenvironment of cutaneous squamous cell carcinoma in organ transplant recipients. *Journal of Immunotherapy*.

[125] Savard M, Bélanger C, Tardif M, Gourde P, Flamand L, Gosselin J (2000). Infection of primary human monocytes by Epstein-Barr virus. *Journal of Virology*.

[126] Shimakage M, Kimura M, Yanoma S (1999). Expression of latent and replicative-infection genes of Epstein-Barr virus in macrophage. *Archives of Virology*.

[127] Croft M (1994). Activation of naive, memory and effector T cells. *Current Opinion in Immunology*.

[128] Salek-Ardakani S, Arrand JR, Mackett M (2002). Epstein-Barr virus encoded interleukin-10 inhibits HLA-class I, ICAM-1, and B7 expression on human monocytes: Implications for immune evasion by EBV. *Virology*.

[129] Haddad E, Paczesny S, Leblond V (2001). Treatment of B-lymphoproliferative disorder with a monoclonal anti-interleukin-6 antibody in 12 patients: a multicenter phase 1-2 clinical trial. *Blood*.

[130] Hinrichs C, Wendland S, Zimmermann H (2011). IL-6 and IL-10 in post-transplant lymphoproliferative disorders development and maintenance: a longitudinal study of cytokine plasma levels and T-cell subsets in 38 patients undergoing treatment. *Transplant International*.

[131] Oertel SH, Riess H (2002). Antiviral treatment of Epstein-Barr virus-associated lymphoproliferations. *Recent Results in Cancer Research*.

[132] Perrine SP, Hermine O, Small T (2007). A phase 1/2 trial of arginine butyrate and ganciclovir in patients with Epstein-Barr virus-associated lymphoid malignancies. *Blood*.

[133] Merlo A, Turrini R, Dolcetti R (2010). The interplay between Epstein-Barr virus and the immune system: a rationale for adoptive cell therapy of EBV-related disorders. *Haematologica*.

[134] Miles JJ, Silins SL, Burrows SR (2006). Engineered T cell receptors and their potential in molecular medicine. *Current Medicinal Chemistry*.

[135] Till BG, Press OW (2009). Treatment of lymphoma with adoptively transferred T cells. *Expert Opinion on Biological Therapy*.

[136] Kennedy-Nasser AA, Bollard CM, Heslop HE (2009). Immunotherapy for Epstein-Barr Virus-related lymphomas. *Mediterranean Journal of Hematology and Infectious Diseases*.

[137] Davis CL, Wood BL, Sabath DE, Joseph JS, Stehman-Breen C, Broudy VC (1998). Interferon-*α* treatment of posttransplant lymphoproliferative disorder in recipients of solid organ transplants. *Transplantation*.

[138] Kahan BD, Yakupoglu YK, Schoenberg L (2005). Low incidence of malignancy among sirolimus/cyclosporine-treated renal transplant recipients. *Transplantation*.

[139] Boratyńska M, Watorek E, Smolska D, Patrzałek D, Klinger M (2007). Anticancer effect of sirolimus in renal allograft recipients with de novo malignancies. *Transplantation Proceedings*.

[140] Mathew T, Kreis H, Friend P (2004). Two-year incidence of malignancy in sirolimus-treated renal transplant recipients: results from five multicenter studies. *Clinical Transplantation*.

[141] Ramachandiran S, Cain J, Liao A (2012). The Smac mimetic RMT5265 · 2HCL induces apoptosis in EBV and HTLV-I associated lymphoma cells by inhibiting XIAP and promoting the mitochondrial release of cytochrome C and Smac. *Leukemia Research*.

[142] Hui EP, Taylor GS, Jia H (2013). Phase 1 trial of recombinant Modified Vaccinia Ankara (MVA) encoding Epstein-Barr viral tumor antigens in nasopharyngeal carcinoma patients. *Cancer Research*.

